# ZAP-X: An Innovative Radiosurgical Solution for Glomus Jugulare Tumors

**DOI:** 10.7759/cureus.86495

**Published:** 2025-06-21

**Authors:** Chengcheng Wang, Jinyuan Wang, Jingmin Bai, Longsheng Pan, Pengfei Xu, Baolin Qu, Xiangkun Dai

**Affiliations:** 1 Department of Radiotherapy, The First Medical Center of the Chinese People’s Liberation Army (PLA) General Hospital, Beijing, CHN; 2 Department of Neurosurgery, The First Medical Center of the Chinese People’s Liberation Army (PLA) General Hospital, Beijing, CHN

**Keywords:** dosimetric comparison, glomus jugulare tumor, long-term follow-up, stereotactic radiosurgery, zap-x

## Abstract

This study evaluated the clinical benefit of the application of the ZAP-X stereotactic radiosurgery (SRS) system in the treatment of glomus jugulare tumors. Two patients with recurrent or progressive glomus jugulare tumors underwent treatment with the ZAP-X SRS system. Analysis of clinical treatment processes and follow-up data over a period of up to three years revealed that after treatment with the ZAP-X system, the tumor volumes significantly reduced, and no severe radiation therapy-related complications were observed. These findings highlight the potential clinical benefits of the ZAP-X system in the treatment of complex skull base tumors.

## Introduction

A glomus jugulare tumor is a rare type of paraganglioma originating from the chemoreceptor cells of the glomus jugulare, located in the jugular foramen. The annual incidence rate of this tumor is approximately one in 1.3 million people [1]. Due to its proximity to critical structures such as the sigmoid sinus, the internal jugular vein, cranial nerves (IX-XII), and the brainstem, surgical resection poses a high risk of neurovascular injury. The rate of complete resection is only 60%-85%, and the incidence of permanent cranial nerve dysfunction after surgery is as high as 20%-50%. Traditional open surgeries, such as the Fisch infratemporal fossa approach, have improved resection rates to 80%-90% through facial nerve transposition and preoperative embolization techniques. However, for patients with recurrence or residual tumors or those who are elderly, more precise minimally invasive treatment strategies are still needed.

The emergence of stereotactic radiosurgery (SRS) and fractionated stereotactic radiotherapy (SRT) has brought about a revolutionary transformation in the treatment of skull base tumors. Clinical studies based on platforms such as the Leksell Gamma Knife and CyberKnife have demonstrated that SRS performs remarkably well in treating glomus jugulare tumors, with five-year local control rates as high as 85%-95%. This result is significantly superior to conventional fractionated radiotherapy, and the rate of cranial nerve injury is below 10% [2-4]. Nevertheless, traditional SRS technologies, due to limitations such as fixed collimator angles, longer treatment times (over 60 minutes per fraction), and inhomogeneous dose distribution, still need to strike a balance between protecting normal tissues and ensuring an adequate tumor dose when dealing with complex targets adjacent to the brainstem or cavernous sinus [5].

The ZAP-X SRS system, as a new-generation precision radiotherapy platform, employs dual-axis rotation technology to achieve submillimeter positioning and irradiation accuracy. It can complete multifractionated treatments within 30 minutes through 200 independently optimized beam fields. The design of its rotating collimator [6], coupled with its self-shielding design, significantly reduces the cost of equipment installation. The MV detector [7] is capable of real-time monitoring of dose accuracy during the treatment process. The smaller penumbra, variety of collimator sizes, and unique treatment planning strategies enable the ZAP-X system to achieve a steeper dose distribution [8]. It can enhance the dose conformity index (CI) of the planning target volume (PTV), significantly outperforming traditional SRS devices [9]. Additionally, the ZAP-X system uses a linear accelerator instead of a radioactive source, thereby improving overall safety and dose rate stability. This study aims to evaluate the dosimetric advantages, tumor control efficacy, and safety of the ZAP-X system in complex skull base targets by analyzing the clinical data of two patients with recurrent/progressive glomus jugulare tumors.

## Case presentation

Clinical presentation

**Figure 1:**
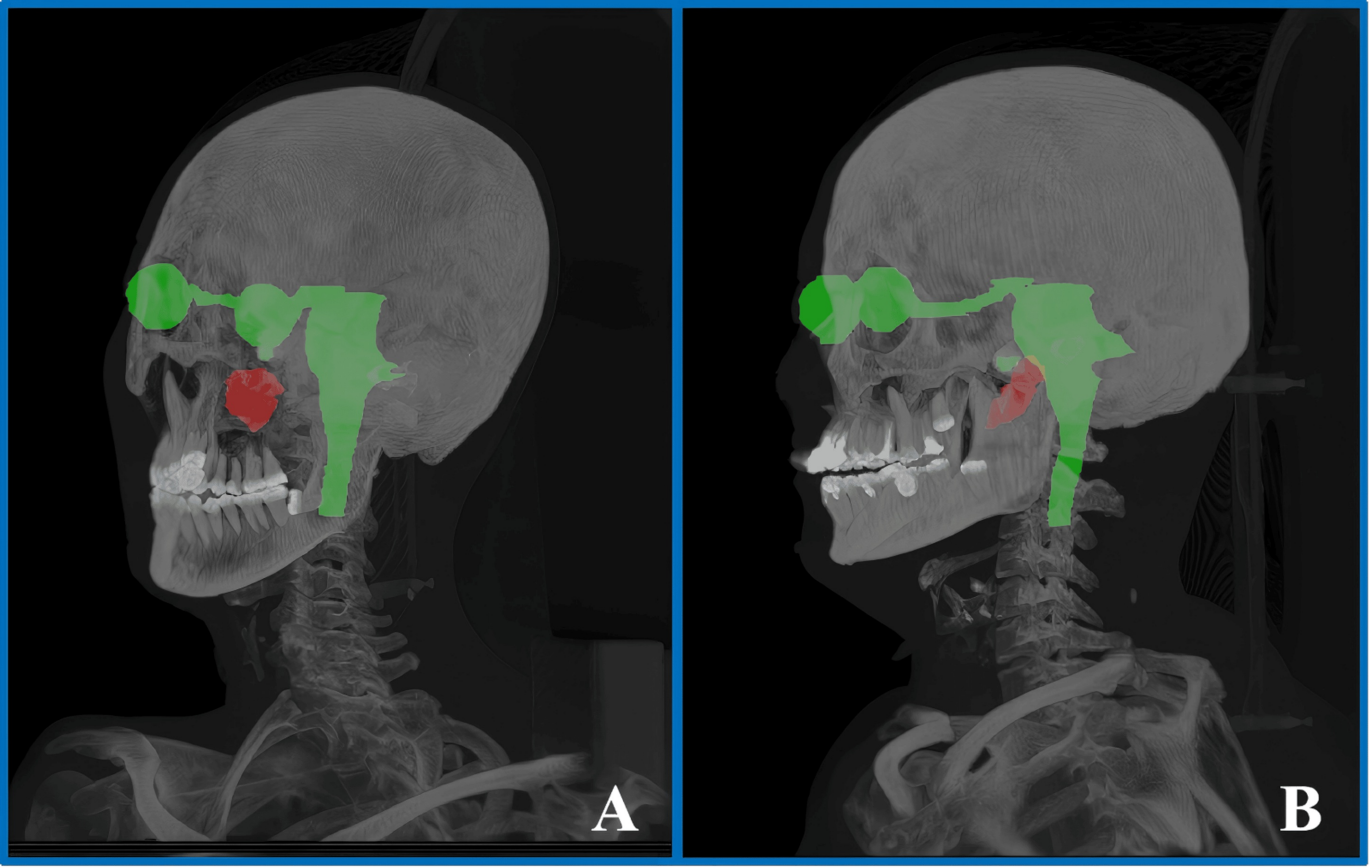
Three-dimensional views of the target locations for the two cases. (A) represents the target location for Case 1, on the patient’s right-hand side of the skull base, and (B) represents the target location for Case 2, on the patient’s left-hand side of the skull base.

Case 1 involved a 38-year-old female patient who presented with symptoms of right-sided hearing loss, hoarseness, and ipsilateral tongue muscle atrophy due to cranial nerve damage in June 2015. There was no apparent cause, and a clear diagnosis was not established at that time. In November 2016, the patient suddenly experienced vertigo, which intensified when her head was turned to the right, accompanied by rotatory nystagmus and projectile vomiting. An enhanced magnetic resonance imaging (MRI) scan revealed an occupying lesion in the right jugular foramen area. On December 13, 2016, the patient underwent tumor resection via a combined occipital-cervical approach, and the postoperative pathology confirmed paraganglioma. During a follow-up in December 2020, an enhanced MRI scan indicated a local recurrence of the tumor. To effectively control the progression of the intracranial recurrent lesion, after thorough communication and discussion with the patient and her family, ZAP-X SRS was performed on the recurrent lesion in January 2021. The lesion was located on the right side of the skull base, as depicted in Figure 1A.

Case 2 involves a 56-year-old female patient who presented with sudden deafness and persistent tinnitus in the left ear in September 2018, with no apparent cause. A high-resolution CT scan of the temporal bone revealed an occupying lesion in the left tympanic cavity and jugular foramen area. On October 30, 2018, a tumor resection of the jugular foramen and tympanic cavity was conducted using a mastoid approach. During the surgery, it was observed that the tumor had encased the jugular bulb and the lower cranial nerves. Given the risk of complete resection causing dysfunction of the lower cranial nerves, a subtotal resection was performed. Postoperative pathology confirmed the presence of a paraganglioma. The patient was monitored with regular follow-up enhanced cranial MRI scans after the surgery. The results indicated that the volume of the residual tumor remained stable from 2019 to 2020. However, in January 2021, a follow-up MRI showed a significant increase in the volume of the irregularly enhanced lesion in the jugular foramen-tympanic cavity complex compared to previous scans, consistent with the imaging characteristics of tumor progression. To effectively control the progression of the residual intracranial lesion, after thorough communication and discussion with the patient and her family, salvage radiosurgery using the ZAP-X SRS system was conducted in January 2021. The lesion was located on the left side of the skull base, as depicted in Figure 1B.

Simulation, delineation, and prescription

All patients were positioned in the supine position. A simulation scan was performed using the Siemens SOMATOM Definition AS CT simulator (Hoffman Estates, IL, USA) with a ZAP-X dedicated positioning board. The CT scan parameters included a slice thickness of 1 mm, a field of view (FOV) of 300 mm, and a scan range extending from the top of the patient’s head (including the head holder) to just above the CT couch. Additionally, magnetic resonance simulation positioning was conducted using the United Imaging Omega MR simulator (Shanghai, China) with a T1+C sequence, also with a slice thickness of 1 mm.

The CT and MR images obtained during positioning were imported into MIM Maestro (version 6.9.5) (MIM Software Inc., Cleveland, OH, USA). After fusing the non-contrast CT with the MR images, the target volumes and organs at risk (OARs) were delineated according to the published consensus guidelines [10]. In this study, the target volumes for both cases included the gross tumor volume (GTV, representing the substantive area of the tumor) and the PTV (expanded by 3 mm from the GTV). The OARs involved in the cases included brain tissue, brainstem, left/right eye, left/right lens, cochlea, left/right optic nerve, optic chiasm, and spinal cord. Both cases were prescribed 21 Gy in three fractions. For the three treatment fractions, dose constraints for the OARs [11,12] are shown in Table 1. The delineated target volumes and CT images were then transferred to the treatment planning system (TPS) of the ZAP-X for plan design.

**Table 1:** Dose constraints for three treatment fractions. OARs: organs at risk

OARs	Dose constraints (3 fractions)
Brainstem	D_max_ < 23.1 Gy, V_15.9_ < 0.5 cc
Optic pathway (including optic nerves and optic chiasm)	D_max_ < 17.4 Gy, V_15.3_ < 0.2 cc
Cochlea	D_max_ < 17.1 Gy
Lens & eye	As low as possible

Treatment planning and dosimetry

The treatment plan was created by a professional physicist with 10 years of experience. The planning criteria are to ensure the PTV coverage is higher than 95%, while minimizing the dose to the OARs, optimizing the CI, and reducing the gradient index (GI) as much as possible. Considering the potential for dose hotspots in high-dose regions, the prescription isodose line is 70%. Our institution believes that this strategy has significant advantages over the conventional 50% normalization method.

**Figure 2:**
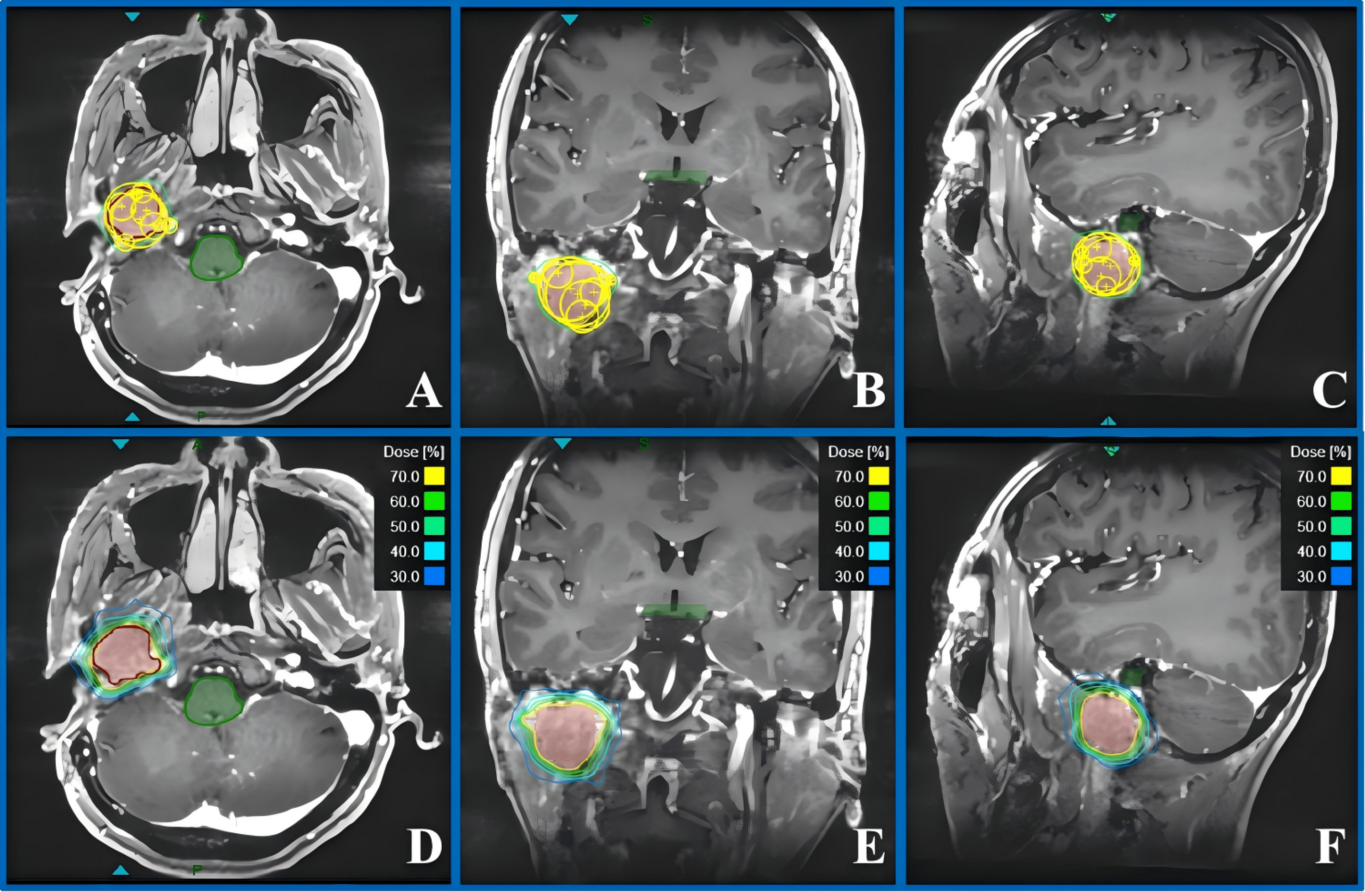
Collimator coverage and dose distribution for Case 1. (A-C) show the collimator coverage of PTV in the axial, coronal, and sagittal planes, respectively. (D-F) show the dose distribution in the axial, coronal, and sagittal planes, respectively. PTV: planning target volume

**Figure 3:**
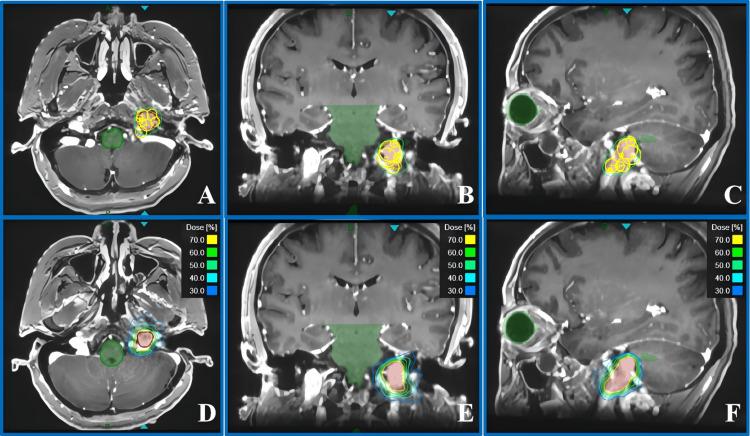
Collimator coverage and dose distribution for Case 2. (A-C) show the collimator coverage of PTV in the axial, coronal, and sagittal planes, respectively. (D-F) show the dose distribution in the axial, coronal, and sagittal planes, respectively. PTV: planning target volume

During the isocenter placement, the automatic isocenter arrangement method (version 1.7.38) of the system tends to result in an excessive number of isocenters. Therefore, we prefer manual placement of the isocenters. We first use a larger collimator to achieve an initial conformal coverage of the target area and then employ smaller collimators to fill in the edges [13]. During dose optimization, inverse optimization algorithms were used. We set constraints for the target volumes and OARs. Ring structures were created to confine the dose around the target, and point doses were set to enhance target coverage. The number of beams is less than 200 to ensure feasible treatment delivery. By analyzing the dose-volume histogram (DVH), the spatial distribution of isocenters was finely tuned, and dose optimization was performed again. This process was repeated until both the target coverage and the dose constraints for OARs met clinical standards; at this point, the final plan step was completed. The final version of treatment plans for both cases achieved good coverage of the target volumes, while the OAR doses were significantly lower than the dose limits. Figure 2 shows the collimator coverage and dose distribution for Case 1, and Figure 3 shows the collimator coverage and dose distribution for Case 2.

**Figure 4:**
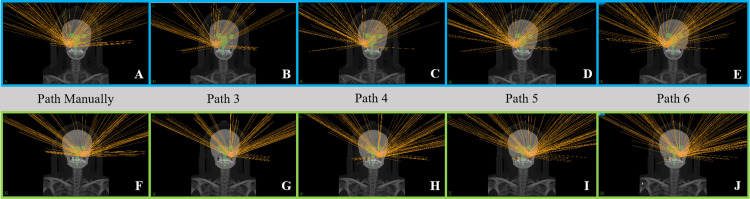
Schematic diagrams of beam distribution for the two cases under different path selections. The images in the top row with blue borders (A-E) show the beam distribution for Case 1, while the images in the bottom row with green borders (F-J) show the beam distribution for Case 2.

Because the TPS version used in our department at the time of treatment was a little old (version 1.7.38), all patients undergoing ZAP-X radiosurgery treatment in our department required a treatment path design before plan creation. Using dedicated software, the initial isocenter was set based on the geometric center of PTV to generate an individualized treatment path for each patient. This ensured a safe distance from the collimator to the patient throughout the treatment process. After the subsequent upgrade of the TPS, the system no longer requires manual design of the treatment path. It came with several general paths, namely, Path 3, Path 4, Path 5, and Path 6. These represent three, four, five, and six evenly distributed circular paths within the available beam space range of 45 to 135 degrees, respectively. The more circular paths there are, the more comprehensive the spatial distribution may be. To compare the impact of the previously manually designed path and the general paths on the plan optimization outcomes and dose distribution, we modified the original plan (manual path) to Path 3, Path 4, Path 5, and Path 6, respectively. We kept the same inverse optimization parameters, as well as the size and position of the isocenters, as in the initial plan, and reoptimized and recalculated the dose for each plan. As shown in Figure 4, these are the beam distribution diagrams for Case 1 and Case 2 under different path selections.

By comparing the five plans for each patient (Table 2), the manually generated path can be replaced by one of the general paths. Therefore, there is no need to worry about the impact of choosing individualized generated paths or general paths for treatment planning. Different paths may be suitable for different target locations, volumes, and shapes. For Case 1, by observing and comparing the target volume parameters of Path 3, Path 4, Path 5, and Path 6, Path 3 and Path 4 have fewer total beams than Path 5 and Path 6. Additionally, the coverage is lower, the D_min_ of PTV is lower, and the GI is higher for Path 3 and Path 4 compared to Path 5 and Path 6. For the OAR parameters, the D_max_ of the bilateral optic nerves and the left eyeball for Path 3 and Path 4 are significantly higher than those for Path 5 and Path 6. However, the D_max_ of the brainstem and cochlea for Path 3 and Path 4 are significantly lower. This may be because increasing the number of beams results in more paths passing through the brainstem and cochlea, but all results are still within the dose limits. The target volume parameters for Path Manually are within the average range of Path 3 to Path 6, and the parameters for OARs are also relatively balanced. For Case 2, the total beams increase with the increased number in the path. Path 3 appears to be the plan with the least favorable target results. However, for the OARs, the doses being higher or lower exhibit a certain degree of randomness, which is related to the target parameter settings during plan design, the optimization method, and the robustness of dose calculation. Nevertheless, all plans can meet the dose constraints at a relatively low level.

**Table 2:** Dosimetric parameters of plans with different paths for the two cases. The conformity index (CI) is calculated as CI = (TVPV × TVPV)/(TV × PV), where TVPV denotes the volume of PTV covered by the prescription dose, TV represents the total PTV volume, and PV signifies the volume encompassed by the prescription dose. The gradient index (GI) is defined as GI = PV50%/PV, where PV50% refers to the volume covered by the 50% prescription dose, and PV is the same as defined for CI [14]. PTV: planning target volume

	Case 1					Case 2				
	Path Manually	Path 3	Path 4	Path 5	Path 6	Path Manually	Path 3	Path 4	Path 5	Path 6
Number of isocenters	12					12				
Collimator chosen	25 mm × 2, 20 mm × 1, 15 mm × 1, 12.5 mm × 2, 10 mm × 1, 7.5 mm × 4, 4 mm × 1					15 mm × 2, 12.5 mm × 1, 10 mm × 3, 7.5 mm × 4, 4 mm × 1				
Total beams	135	87	113	141	149	131	113	133	148	145
CI	1.2	1.17	1.18	1.22	1.21	1.33	1.29	1.26	1.23	1.25
GI	2.84	2.91	2.8	2.68	2.7	3.44	3.58	3.38	3.43	3.33
Coverage (%)	97.51	93.7	95.92	98.13	97.96	96.97	92.97	95.35	94.19	96.54
PTV D_min_ (cGy)	1,785.9	1,568.45	1,695.2	1,819.12	1,838.67	1,847.03	1,822.73	1,880.86	1,893.57	1,923.28
Right optic nerve D_max_ (cGy)	826.48	646.24	577.89	484.96	481.99	155.13	372.55	440.37	83.17	119.53
Left optic nerve D_max_ (cGy)	680.64	651.91	639.75	509.35	574.33	222.27	144.36	356.75	264.16	298.53
Right lens D_max_ (cGy)	44.42	42	41.59	66.41	52.46	53.22	54.57	181.23	759.84	440.81
Left lens D_max_ (cGy)	174.05	726.2	45.81	322.02	52.07	143.97	420.34	128.19	223.17	130.3
Right eye D_max_ (cGy)	439.14	304.21	295.7	307.9	314.29	418.75	852.94	755.41	760.9	494.99
Left eye D_max_ (cGy)	353.63	918.1	703.61	595.46	444.41	150.52	655.67	465.48	234.78	250.76
Optic chiasm D_max_ (cGy)	888.22	545.07	692.06	699.3	792.39	879.28	638.08	672.5	685.61	862.07
Brainstem D_max_ (cGy)	447	276.54	420.15	663.92	608.8	1,248.24	1,118.7	1,270.81	1,348.26	1,360.81
Cochlea D_max_ (cGy)	972.33	737.37	890.18	1,051.8	1,008.17	1,528.82	1,491.25	1,408.69	1,002.83	1,420.52
Spinal cord D_max_ (cGy)	266.25	225.35	222.66	275.144	241.66	191.76	268.29	244.51	271.52	351.39

Follow-up results

In this study, the two patients had tumor volumes of 6.98 and 3.43 cc, respectively. When undergoing SRS using the ZAP-X system, the average treatment duration from patient positioning to the end of treatment for the two patients was 27.33 minutes per fraction and 28.07 minutes per fraction, respectively.

**Figure 5:**
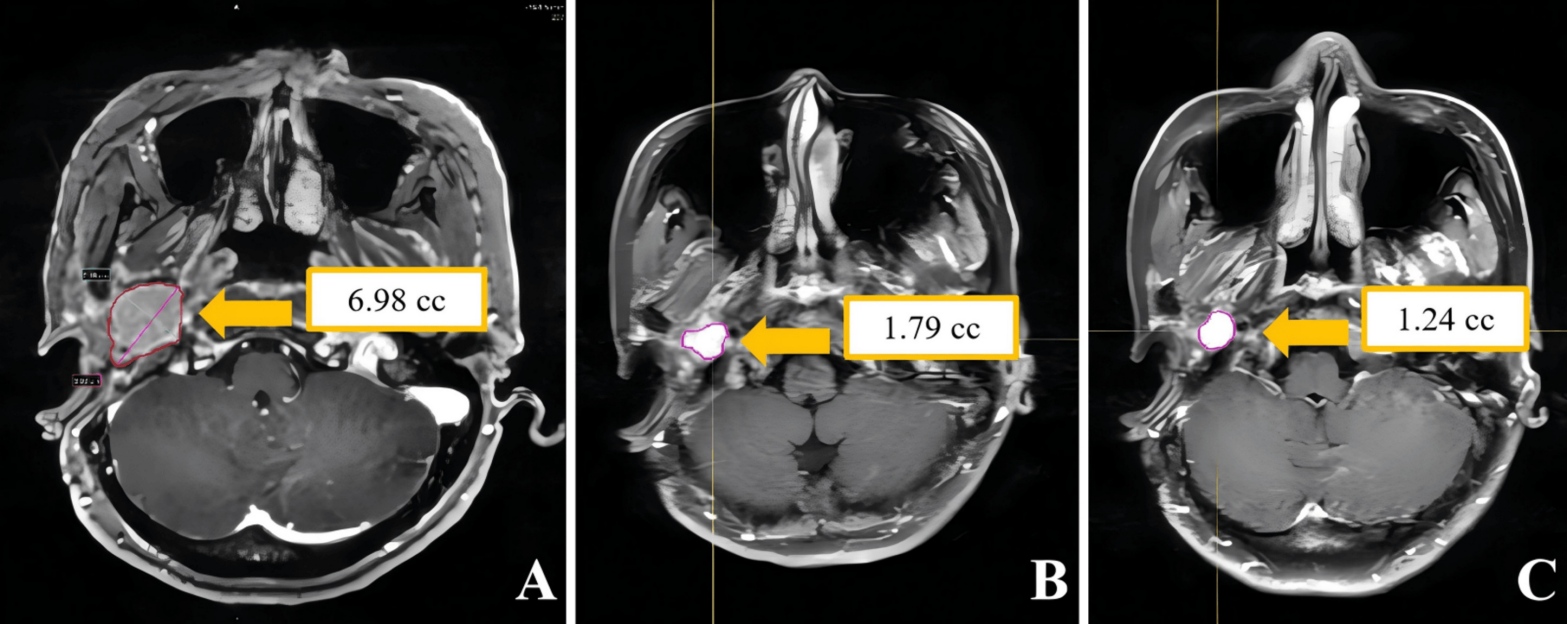
Contrast-enhanced MRI images of Case 1. (A) shows axial contrast-enhanced T1-weighted imaging with contouring of the tumor volume before radiosurgery; (B) shows the same sequence with tumor volume contouring one year post-ZAP-X radiosurgery; (C) shows the axial contrast-enhanced T1-weighted imaging with tumor volume contouring three years post-ZAP-X radiosurgery.

In Case 1, no acute radiation therapy-related toxicities of Common Terminology Criteria for Adverse Events (CTCAE) grade ≥ 2 were observed throughout the entire treatment course. For follow-up results, a gadolinium-enhanced MRI assessment conducted one year after treatment showed a reduction in tumor volume from a baseline of 6.98 to 1.79 cc (a 74.4% decrease in volume), and the MRI-T2 sequence did not reveal any peritumoral edema or radiation necrosis. Continuous follow-up to three years post-treatment revealed further volume reduction to 1.24 cc (cumulative volume reduction reached 82%), which met the partial response criteria according to the Response Assessment in Neuro-Oncology (RANO) standards. During the long-term follow-up period, no significant new neurological deficits or treatment-related toxicities were observed. The radiological evolution of the tumor region in Case 1 is detailed in Figure 5.

**Figure 6:**
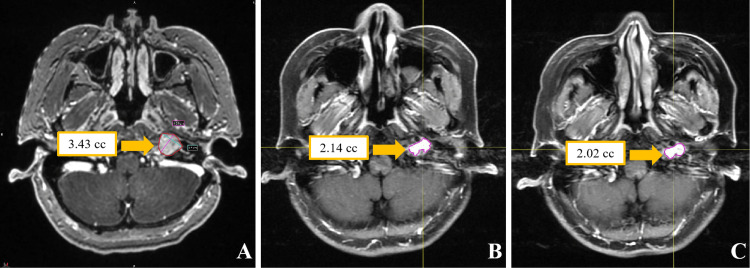
Contrast-enhanced MRI images of Case 2. (A) shows an axial contrast-enhanced T1-weighted image with contouring of the tumor volume before radiosurgery; (B) shows the same sequence with tumor volume contouring one year post-ZAP-X radiosurgery; (C) shows the axial contrast-enhanced T1-weighted imaging with tumor volume contouring three years post-ZAP-X radiosurgery.

The patient in Case 2 did not experience any acute radiation therapy-related toxicities of CTCAE grade ≥ 2 during the treatment period. A gadolinium-enhanced MRI assessment conducted one year post-treatment showed a reduction in tumor volume from a baseline of 3.43 to 2.14 cc (a volume reduction rate of 37.6%), and the MRI-T2 sequence did not reveal any signs of radiation-induced cerebral edema. Continuous follow-up to three years post-treatment revealed a further reduction in tumor volume to 2.00 cc (cumulative volume reduction of 41.7%). Radiological assessment using the RANO criteria confirmed that the disease had reached a stable state. During the long-term follow-up period, neurological examinations did not reveal any new positive signs, and treatment-related toxicity assessments showed no adverse reactions of CTCAE grade ≥ 2. Figure 6 illustrates the radiological evolution of the tumor region in Case 2.

## Discussion

The ZAP-X SRS system, with its innovative technical architecture, provides a precise treatment option for complex skull base tumors that are located near critical neurovascular structures, such as glomus jugulare tumors. Its core advantages are mainly reflected in three aspects: First is the precision of dosimetry. Through the dual-axis rotational focusing technology and 200 independently optimized beams, it achieves high conformal dose coverage of irregular targets. In this study, the PTV coverage for the two patients reached 97.51% and 96.97%, respectively, with CI of 1.20 and 1.33. This is in agreement with the previous research findings of our institution that the ZAP-X system has shown superior target coverage and a steep dose gradient around the target, significantly better than traditional SRS devices in the treatment of cerebral lesions [15]. Second is the improvement in treatment efficiency. With the 6D skull tracking technology and real-time motion correction, the single fraction treatment time was approximately 28 minutes, which is more than 50% shorter than traditional Gamma Knife, significantly improving patient comfort. The third is the protection of normal tissues. Through the treatment planning strategy of "using large-aperture collimators to quickly cover the main target volume + small-aperture collimators to optimize edge conformity" [13], the dose received by OARs, such as the brainstem, optic nerves, and cochlea, is far below the safety threshold, and no treatment-related toxicities of CTCAE grade ≥ 2 have been observed. This confirms the safety of ZAP-X in complex anatomical regions.

Regarding treatment efficacy, the two patients demonstrated distinct patterns of tumor control: Case 1, a patient with postoperative recurrence, experienced an 82% reduction in tumor volume within 36 months post-treatment, resulting in partial remission; Case 2, a patient with disease progression after subtotal resection, saw a 41.7% reduction in tumor volume within the same period post-treatment, maintaining a stable disease state. This variance may be attributed to the initial tumor burden (6.98 cc versus 3.43 cc) and dose constraints of adjacent structures. Importantly, during long-term follow-up, neither patient reported any deterioration in cranial nerve function, underscoring the significant advantage of ZAP-X in neuroprotection, which is attributed to its submillimeter dose precision and steep dose gradient [8].

In our study, direct comparison was limited due to sample size and case variability, but Yazici et al. [4] found that while SRT demonstrated an excellent local control rate for head and neck (H&N) paragangliomas, tumor volume reduction may last beyond the initial year of treatment in a substantial proportion of patients; this also gave confidence for our three-year follow-up results. Through the follow-up observation of up to three years for the two patients, we preliminarily found that the local control rate after ZAP-X treatment was comparable to that reported in traditional SRS studies, and patients' quality of life was significantly improved. Ong et al. [16] analyzed 23 studies involving 460 patients with glomus jugulare tumors treated with SRS, with a mean follow-up of 47 months, concluding a 95% tumor control rate and 47% clinical symptom improvement. Ehret et al. [1] evaluated outcomes in 101 glomus jugulare tumor patients, including 88 treated with single-dose radiotherapy (median dose 16 Gy) and 13 with up to five fractions (median dose 21 Gy). With a median follow-up of 35 months, the overall local control rate was 99%, and 56% of patients showed symptom improvement or complete resolution. Although long-term efficacy data on ZAP-X for the treatment of glomus jugulare tumors are still relatively limited at present, and the control group is lacking, such as Gamma Knife or CyberKnife-treated patients, the preliminary results of this study provide important references for subsequent clinical applications. These results indicate that the ZAP-X system not only is innovative in technology but also demonstrates significant clinical advantages and is expected to become an important tool for the future treatment of glomus jugulare tumors.

However, despite the promising preliminary outcomes and application prospects of the ZAP-X system in the treatment of glomus jugulare tumors, further clinical research such as larger prospective studies is still needed to comprehensively validate its long-term efficacy and safety. Future studies should include more cases and extend the follow-up period to evaluate the therapeutic effects of the ZAP-X system. In addition, further optimization of treatment planning and dose distribution, as well as exploration of the combination of ZAP-X with other treatment modalities (such as surgery and chemotherapy), will also be important directions for future research. We look forward to these in-depth studies to provide more precise and effective treatment options for patients with glomus jugulare tumors, thereby further improving their prognosis and quality of life.

## Conclusions

In this study, we have detailed the entire process of SRS using the ZAP-X system for two patients with glomus jugulare tumors. By analyzing the clinical treatment processes and follow-up data over a period of up to three years for these two patients, we found that after receiving ZAP-X treatment, the patients' symptoms were significantly alleviated, the tumor volumes continued to shrink during the follow-up period, and no severe radiation therapy-related complications such as cranial nerve injury, brainstem injury, or cerebrospinal fluid leak were observed. These results fully demonstrate the significant potential advantages of the ZAP-X system in the treatment of glomus jugulare tumors, especially in protecting the critical neurovascular structures surrounding the target area in brain tissue. Additionally, the high-precision dose distribution and flexible beam angle design of the ZAP-X system enable it to better accommodate tumors with complex shapes, further enhancing the safety and efficacy of the treatment. The results of this study provide important clinical references and reliable evidence for the application of ZAP-X radiosurgery in the treatment of complex skull base tumors such as glomus jugulare tumors and hold the promise of offering more safe and effective treatment options.
